# Atypical Central Neurocytoma with Recurrent Spinal Dissemination over a Period of 20 Years: A Case Report and Review of the Literature

**DOI:** 10.1155/2013/925647

**Published:** 2013-06-09

**Authors:** Tareq A. Juratli, Kathrin Geiger, Mario Leimert, Gabriele Schackert, Matthias Kirsch

**Affiliations:** ^1^Department of Neurosurgery, University Hospital Carl Gustav Carus Dresden, Technical University of Dresden, Fetscherstrasse 74, 01307 Dresden, Germany; ^2^Institute of Neuropathology, University Hospital Carl Gustav Carus Dresden, Technical University of Dresden, Fetscherstrasse 74, 01307 Dresden, Germany

## Abstract

We present an unusual case of a late recurrent central neurocytoma that was rediagnosed as an ependymoma and neurocytoma in accordance with changes in histological classifications. *Case Description*. A 56-year-old male teacher presented with incomplete transverse syndrome due to several intradural extramedullary tumors at the level of lumbar vertebrae 1–3. The histological diagnosis at the time was atypical ependymoma. One year later, two additional tumors were removed at the L5-S1 vertebral level. For 12 years, the patient remained tumor free on followup. Fourteen years after the initial diagnosis, the patient presented with thoracic paresthesias due to two new extramedullary tumors in the C7-T1 and the T8-T9 vertebral levels. After complete removal of the tumors, a radiological survey revealed an intracranial lesion in the third ventricle. Five months later, an additional lesion recurrence was removed surgically. The most recent histological diagnosis revealed an atypical central neurocytoma. In retrospect, the previous tumors were reclassified as neurocytoma according to the additional immunohistochemistry evidence. *Discussion*. There is no standard adjuvant treatment regimen for atypical neurocytoma; therefore, the patient is currently under close followup. Modern histopathological diagnosis is essential in these cases. Potential routes for dissemination of the tumor should be considered upon first recurrence.

## 1. Introduction

Central neurocytomas are rare benign tumors of the central nervous system, characterized by their intraventricular localization. They are considered to arise from precursor cells of the septum pellucidum. They predominantly occur in young adults and generally have a favorable outcome, although cases with an aggressive clinical course and recurrences have been described. Historically, many of these lesions were regarded as either intraventricular oligodendroglioma or as ependymoma until detailed immunohistological clarification of their neuronal phenotypes was established. Neurocytoma was first described by Hassoun et al. [1] and is now a well-established diagnosis included in the latest WHO Classification [2]. 

In the literature, only a few neurocytomas were reported with an extraventricular location, including cerebral hemispheres [3], thalamus [4], cerebellum [5], pons [6], amygdala [7], retina [8], and in some rare cases the spinal cord [9]. 

Herein, we report a case of an atypical central neurocytoma with recurrent spinal dissemination over a period of 20 years.

## 2. Case Report 

A 56-year-old Caucasian male teacher was first seen by his general practitioner in September 1991 complaining of back pain that was treated symptomatically. No imaging studies were arranged at that time. In April 1992, the patient developed sudden paraplegia of both legs, necessitating emergency surgery in an outside hospital. After a myelography and post-myelography-CT, three tumors located in the L1-3 vertebral level were removed via laminectomy L1-3. The paraplegia was caused by bleeding within the biggest tumor at the L1 level. Investigation of the removed tumors showed an “atypical” ependymoma. Immunohistochemical tests were not performed at that time. After removal, the neurological symptoms persisted, including saddle anesthesia, sphincter disturbance (urinary retention and fecal incontinence), and weakness of both legs. 

In December 1992, a second operation (also in an outside hospital) was necessary to remove two newly appeared tumors which were located in the L5-S1 vertebral level. The histological study of the removed tumors also demonstrated an atypical ependymoma. Since that time, the patient has had no new clinical presentations. However, the symptoms mentioned previously persisted.

**Figure 1:**
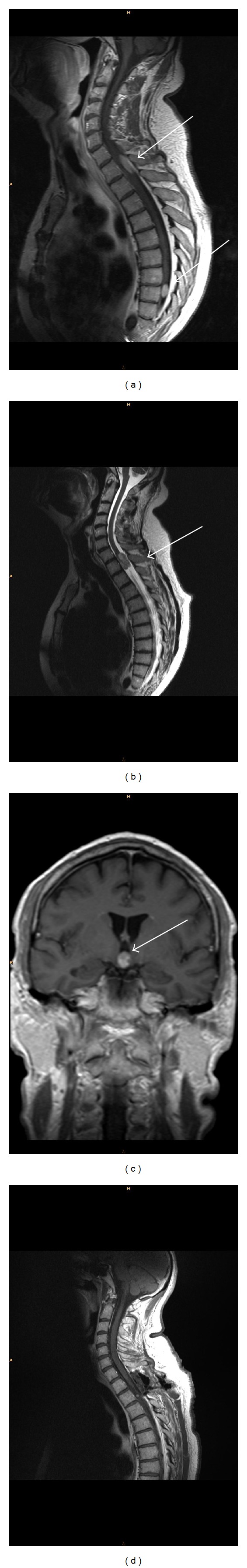
(a) A sagittal postgadolinium T1-weigthed image shows the two lesions before surgery. (b) The tumor at level C7-T1 in T2-weigthed image. (c) In cranial T1-weighted image, the small tumor is located on the floor of the third ventricle. (d) T1-weighted image shows the postoperative result.

In December 2006, the patient was admitted to our neurosurgical department because of numbness and pain radiating into the upper part of the body and into both arms. The spinal MR imaging showed several intramedullary spaces occupying lesions in the C7-T1 and the T8-T9 vertebral levels (Figures 1(a) and 1(b)). The large lesion at the level of C7-T1 was completely removed via laminectomy. Intraoperatively, the tumor appeared intradural extramedullary with direct contact to the left T1-radix. Complete removal was accomplished without causing any postoperative neurological morbidity. In March 2007, removal of the tumor located at the T8-T9 vertebral level was undertaken (Figure 1(d)). Again, the tumor was extramedullary, originating from a spinal nerve within the cauda equina. Postoperatively, the patient did not suffer from any complaints and no neurological worsening was observed. 

Due to repeated presentations of multiple ependymoma, a cranial MRI was obtained for the first time. This revealed a contrast enhancing intracerebral mass in the third ventricle (Figure 1(c)). The cranial mass did not cause any symptoms. In particular, no visual field deficits or hormonal disturbances were detectable. The patient refused any surgical intervention to treat the cranial tumor and preferred close follow-up investigations.

In the MR imaging studies of the cranium and spinal axis in September 2010 and 2012, no new tumors were seen and progression of the cranial tumor was indiscernible.

## 3. Histology

**Figure 2:**
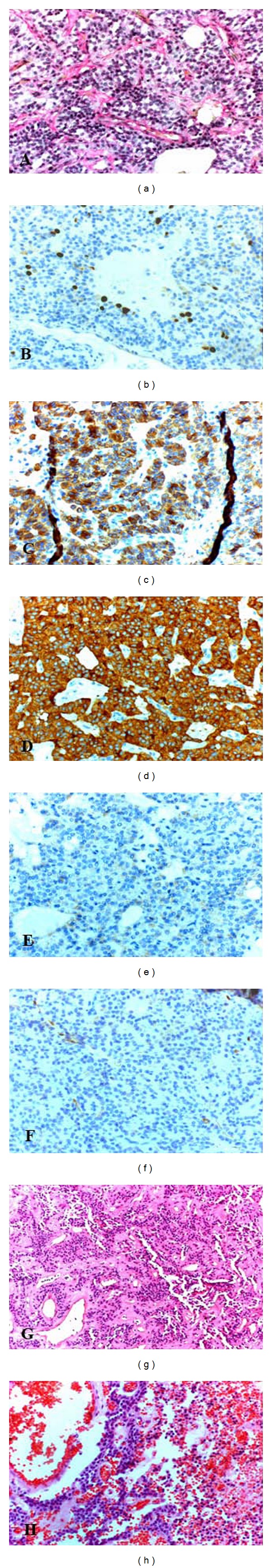
Tumor histomorphology at different time points. (a) In 2007, excised mostly monomorphic highly cellular tumor with regular rounded nuclei and only few mitoses. The blood vessels are regularly arranged resembling a loose meshwork without pathological endothelial proliferation or glycogen storage. (Periodic acid Schiff (PAS), Magnification original ×20.) (b) Immunostaining for the proliferation marker K67 (MIB-1) showing only few proliferating cells (indirect peroxidise technique (LSABII, DAKO, diaminobenzidine (DAB) as a chromogen, brown, Magnification orig. ×20, counterstaining with Hematoxylin)). (c) Immunostaining for *β*-III-Tubulin (Chemicon), a marker of immature neural cells with strong positivity of all tumor cells. (LSABII, DAKO, (DAB), counterstaining with Hematoxylin Magnification orig. ×20.) (d) Immunostaining for the neuronal marker synaptophysin (Dako) with strong positive staining of all tumor cells. (LSABII, DAKO, (DAB), counterstaining with Hematoxylin Magnification orig. ×20.) (e) Immunostaining for chromogranin (Dako), a marker of highly differentiated neuronal cells and ganglion cells with mostly negative staining results. (LSABII, DAKO, (DAB), counterstains with Hematoxylin Magnification orig. ×20.) (f) Immunostaining for S100 (Dako) showing a mostly negative staining result with single positive cells of glial morphology at irregular intervals (LSABII, DAKO, (DAB), counterstaining with Hematoxylin Magnification orig. ×20.) (g) Tumor biopsy of 2006 with similar morphology of lower cellularity. Noteworthy are the occasional cell-free areas. (H&E, Magnification original ×10.) (h) Tumor biopsy of 1992 showing similarly organized tumor cells with rounded nuclei and even less mitoses within an extensive hemorrhage. (H&E, Magnification original ×20.)

Histology demonstrated a mostly monomorphic, highly cellular tumor with rounded chromatin intense nuclei, mostly without nucleoli or mitoses (Figures 2(a) and 2(b)). Nuclear pleomorphy was low and was lacking atypical mitoses. A regular rete-like network of otherwise inconspicuous capillaries was distributed throughout the tumor. Rosettes of fully differentiated ganglion cells were absent. Likewise, the intratumoral matrix showed only mild fibrillary content with marked absence of typical glial fiber networks. The walls of larger blood vessels appeared slightly hyaline with thickened ends. Inflammation was mostly absent. Immunohistochemistry revealed a strong positive reaction for the neuronal marker synaptophysin and for the antibody to *β*III Tubulin (Figures 2(c) and 2(d)) demonstrating mostly immature neurons in 100% of the tumor cells. Chromogranin, a marker for mature, differentiated neurons and ganglion cells, was mostly negative (Figure 2(e)). GFAP (not shown) and S100 were mostly negative with the exception of single cells of glial morphology (Figure 2(f)). Only few proliferating cells (up to 5% maximum) could be ascertained with the marker Ki67/MIB-1 (Figure 2(b)). The tumor was evaluated as atypical neurocytoma (equivalent to WHO grades II-III) due to increased cellularity and slightly elevated counts of proliferating cells. An ependymoma could be mostly excluded on the basis of lacking GFAP stain. In comparison, the tumor tissues excised in 2006 showed a similar tumor pattern, with slightly lower cellularity and perivascular nucleus-free neuropil, typical of neurocytoma (Figure 2(g)). Immunohistochemical evaluation also showed the same expression pattern as the tumor from the surgery in 2007, with a positive immunoreaction for synaptophysin and a negative reaction for GFAP. Slides of the tumor excised in 1992 also showed a similar morphology (Figure 2(h)) and a similar reaction pattern, with a slightly lower proliferation rate as compared to 2007, and a negative immunoreaction for GFAP (not shown). In retrospect, both tumor tissues were reclassified as neurocytoma, which did not show atypical morphology in both instances.

## 4. Discussion 

Central neurocytomas are benign tumors of the central nervous system which predominantly occur in young adults and generally have a favorable outcome. Neurocytomas are typically located in the lateral ventricles in the region of foramen Monro (central neurocytoma) or within the brain parenchyma (extraventricular neurocytoma). 

Atypical neurocytomas, which are neurocytomas with a MIB-1 LI >2% with/without anaplastic features [15, 16], have been noted to have worse clinical outcome and higher recurrence rate compared with normal neurocytomas [16, 17]. Söylemezoglu et al. observed that patients with neurocytoma MIB-1 LI <2% had a relapse rate of 22% after a followup of 150 months compared with 63% when it was >2%. Thus, they concluded that a MIB-1 LI >2% might be critical in determining recurrence. Rades et al. have suggested that a MIB-1 LI score >3% is associated with an adverse outcome [18]. In our case, the last removed tumor in March 2007 exhibited a MIB-1 LI of 5%. 

Despite the usually benign nature of this tumor, eight cases with ventricular or spinal dissemination have been reported in the literature (Table 1). Sharma et al. reported a rare case of a spinal neurocytoma with a retrograde caudo-cranial CSF dissemination in a 24-year-old patient [9]. The dissemination pattern of the presented case was difficult to investigate since cranial imaging studies were not performed upon initial presentation. In our case, it remains difficult to determine whether the patient actually suffered a retrograde dissemination of the tumor or whether the tumor originally consisted of a small central neurocytoma with long-term spinal dissemination through the ventricular system. One potential argument in favor of the latter notion is the fact that, in 2006, the previous tumor showed the typical morphology of a central neurocytoma without atypia and may have grown very slowly over time. Atypical behavior clearly only developed recently. Although not obviously malignant, the biological behavior of this tumor has to be regarded as uncertain.

Pitfalls of the pathological diagnosis consist of morphology very similar to ependymoma, which also contains perivascular acellular areas, but subtly fewer monomorphic nuclear shapes and a more fibrillar matrix. In this case, the first diagnosis of an ependymoma which is much more likely in this location was based only upon morphology. However, using immunohistochemistry, it was possible to reach the correct diagnosis of neurocytoma. Paraganglioma represents a further differential diagnosis of spinal neurocytoma. Immunohistochemical differentiation between these two entities is considerably more difficult since both tumors show expression of neuronal markers; however, paraganglioma should also show expression of chromogranin and at least some more mature neural cells. Furthermore, paraganglioma usually contains a morphological “Zellballen” pattern and sustentacular cells at regular intervals with positive staining of S100 [19]. These specific features are absent in the tumor described in this report. Also, the clinical course of our patient with intraspinal localization of the tumors and the intracerebral lesion makes a paraganglioma very unlikely in this case. A further argument towards the neurocytoma is the slightly different morphology of the previous tissue obtained in 2006 which shows even more clearly defined aspects of a neurocytoma. The age of the patient (42 years at first appearance of symptoms) is slightly above the median age in neurocytoma patients, which occurs mainly in young adults. The recurrence and dissemination pattern over 10 years is also highly unusual.

In summary, the case is of interest to the readership due to its unusual course, intracerebral spread, and long duration with only incidental pathology, as well as its ambiguous histological interpretation over the years and neuropathological classification. Early cranial imaging studies are considered upon repeated spinal recurrences to exclude a primary “seeding” tumor.

## Figures and Tables

**Table 1:** Reported cases of neurocytoma with dissemination.

Case number	Authors and year	Location of the primary tumor	MIB-1 LI (%)	Location of the dissemination
1	Sharma et al., 2005 [9]	C5-T1 spinal segments	9%	Intraparenchymal cerebellar
2	Takao et al., 2003 [10]	Lateral ventricle	4.6%	Local and spinal dissemination
3	Brandes et al., 2000 [11]	Septum pellucidum	Unknown	Ventricular and spinal dissemination
4	Elek et al., 1999 [12]	Lateral ventricle	4.4%	Local, ventricular, and leptomeningeal dissemination
5	Eng et al., 1997 [13]	Septum pellucidum	3.3%	Local, ventricular, and spinal dissemination
6	Eng et al., 1997 [13]	Lateral ventricle	1.8%	Local and leptomeningeal dissemination
7	Tomura et al., 1997 [14]	Lateral ventricle	Unknown	Ventricular dissemintaion
8	Tomura et al., 1997 [14]	Lateral ventricle	Unknown	Local and ventricular dissemination

MIB-1 LI: MIB-1 Labeling index.
